# Editorial: Insights into fungal biology with emphasis on pathogenesis in humans

**DOI:** 10.3389/ffunb.2024.1438060

**Published:** 2024-07-02

**Authors:** Angel Gonzalez, Marilene Henning Vainstein, Patrícia Albuquerque, Ildinete Silva-Pereira

**Affiliations:** ^1^ Basic and Applied Microbiology Research Group (MICROBA), School of Microbiology, Universidad de Antioquia, Medellín, Colombia; ^2^ Centro de Biotecnologia, Universidade Federal do Rio Grande do Sul, Porto Alegre, RS, Brazil; ^3^ Laboratory of Molecular Biology of Fungi, University of Brasilia, Brasilia, Brazil

**Keywords:** fungal virulence, pathogenic fungi, antifungal drugs, fungal biology, research topic

Fungi are exotic and fantastic microorganisms that thrive in the most contrasting environments, establishing many of ecological interactions. Depending on the nature of their interactions with humans or other animals, fungi can be viewed as friends or foes. This dual nature has aroused considerable interest in studying human pathogenic fungi driven by three pillars: the alarming level of fungal diseases of worldwide, the limited efficacy of current antifungal drugs, and the increasing incidence of antifungal resistance. In response to these challenges, the World Health Organization (WHO) has recently proposed a list of 19 fungal pathogens of critical, high, or medium priority based on criteria such as incidence, mortality, drug resistance, and treatment options. This aims to guide research efforts and policy interventions to strengthen the global response to fungal infections and antifungal resistance (WHO, 2022).

This Research Topic includes several reports addressing molecular biology related to the fungal-host interplay. Studies explore transcriptomic reprograming, the host immune response upon fungal infection, and the mechanisms by which fungi evade the host defenses using their repertoire of virulence attributes. Noteworthy, among the fungal pathogens listed by the WHO are *Candida albicans*, *Aspergillus fumigatus*, and *Pneumocystis jirovecii*, three of the pathogenic fungi that most frequently cause fungal infections worldwide, which are the object of study in this Research Topic.

The increasing availability of complete microbial genomes in databases has led to significant advances allowing the detailed study of many genes using this valuable information. However, these databases often have some read-length limitations (i.e., < 1 kb) (Eid et al., 2009); therefore, advances in DNA sequencing technology now support the generation of highly accurate long-read data sets (i.e., > 20 kb). For instance, in *Candida albicans*, while resources are highly developed, published sequences may contain gaps and may not extend to the telomeres on its chromosomes. Moreover, genome assembly is complicated by extensive repeated sequences and allelic variation. In these lines, Hoyer et al. have developed a new in silico method derived from a PacBio HiFi data set, facilitating telomere-to-telomere genome assembly and accurate sequencing of several genes. This proposed new methodology promises to enhance the study of large and complex genes in *Candida* and other medical-importance fungi.

Historically considered a commensal fungus in mammals, there is increasing evidence that *Candida albicans* inhabit several environmental niches (Opulente et al., 2019; Sautour et al., 2021). This could indicate that *Candida* can adapt to many environments, allowing it to enhance its pathogenicity. In this sense, it has been proposed that free-living amoebas can control fungal populations in the soil, exerting predator selection pressure that might have influenced the evolution of virulence attributes of several fungi, including *Cryptococcus neoformans*, *Aspergillus* spp., and *Candida* spp. (Casadevall et al., 2019). The study conducted by Amsri et al. investigated *C. albicans* during interactions with *Acanthamoeba castellanii* and found that fungal exposure to amoebae enhanced *Candida* survival’s, induced visible morphological changes, as well as increased its fitness to several stressors, thereby increasing its virulence factors. These findings suggest that protozoa interaction may drive microevolutionary changes in fungal pathogens, modulating their ability to change their phenotypes and virulence factors.

Pulmonary infections caused by *Aspergillus fumigatus* are a leading cause of morbidity and mortality worldwide, responsible for up to 90% of all cases of pulmonary aspergillosis (Perfect et al., 2001; Latge and Chamilos, 2019). This pathogen is commonly isolated from patients with cystic fibrosis (CF) (Warris et al., 2019). In this patients, mutations in the CF transmembrane conductance regulator gene (CFTR) result in bronchial epithelial dysfunction, which includes dysregulation of epithelial electrolyte flux and build-up of hyperviscous mucus, increasing the risk of bacterial and fungal respiratory infections (Gadsby et al., 2006; Kreda et al., 2012). Illek et al. investigated the roles of different morphotypes of *A. fumigatus* (conidia, germlings, and hyphae) and its virulence factor gliotoxin on epithelial integrity in CFTR mutant in comparison with a wild-type bronchial epithelial cell line. They observed that conidia were more internalized by mutant bronchial cells; and during fungal morphogenesis, bronchial cells showed signs of damage, an effect that was faster when employing hyphae and gliotoxin than with conidia and germlings. The authors suggest that the growth and internalization of *A. fumigatus* result in deleterious effects on bronchial epithelial barrier function that occurred more rapidly in the absence of CFTR, indicating a protecting role of this gene supporting the mucociliary clearance and delaying the loss of epithelial integrity during fungal infection.

In the last decade, three-dimensional (3D) culture techniques including organoid technology, have advanced significantly, allowing the generation of rudimentary organ-like structures and the study of microbial pathogenesis among other processes (Akkerman and Defize, 2017). In the present Research Topic, Tisdale-Macioce et al. reported the development of a murine lung organoid that mimics the lung alveolar sacs composed of alveolar type 1 and type 2 cells. This model could be used to study many aspects of the biology of the *Pneumocystis* spp, particularly *P. jirovecii* which causes pneumonia, mainly in immunosuppressed individuals (Higashi et al., 2017). Notably, as this fungal pathogen cannot be grown in conventional mycological culture media, this organoid model represents a valuable tool for studying its biology and interactions, in addition to opening the possibilities to study the host-pathogen interactions with other bacterial, viral, or fungal pathogens.

Finally, Pinto et al. reported the first isolation of *Hanseniaspora opuntiae* from four pregnant women in Brazil. Notably, this fungus was isolated as part of the routine ante-natal care for maternal colonization screening for *Streptococcus agalactiae* group B. Although this fungus has not been associated with infection in humans, causing a specific pathology or any symptomatology, this finding should be explored to identify it as part of vaginal microbiota or as a potential emerging fungal pathogen in pregnant or immunosuppressed populations.

The contributions described in this Research Topic highlight recent advances in understanding fungal pathogen biology and their interactions with humans and other environmental hosts. Additionally, these works emphasize the importance of implementing new study models such as cell cultures or organoids, for exploring these interactions as well as evaluating new therapeutic strategies to deal with these fungal affections.

## Author contributions

AG: Conceptualization, Writing – original draft, Writing – review & editing. MV: Writing – review & editing. PA: Writing – review & editing. IS: Writing – review & editing.
